# High mRNA expression of splice variant *SYK* short correlates with hepatic disease progression in chemonaive lymph node negative colon cancer patients

**DOI:** 10.1371/journal.pone.0185607

**Published:** 2017-09-28

**Authors:** Robert R. J. Coebergh van den Braak, Anieta M. Sieuwerts, Raju Kandimalla, Zarina S. Lalmahomed, Sandra I. Bril, Anne van Galen, Marcel Smid, Katharina Biermann, J. Han J. M. van Krieken, Wigard P. Kloosterman, John A. Foekens, Ajay Goel, John W. M. Martens, Jan N. M. IJzermans, on behalf of the MATCH study group

**Affiliations:** 1 Department of Surgery, Erasmus MC University Medical Center, Rotterdam, the Netherlands; 2 Department of Medical Oncology, Erasmus MC Cancer Institute, Erasmus MC University Medical Center, Rotterdam, the Netherlands; 3 Cancer Genomics Center Netherlands, Amsterdam, The Netherlands; 4 Center for Gastrointestinal Research and Center for Epigenetics, Cancer Prevention and Cancer Genomics, Baylor Scott and White Research Institute and Charles A Sammons Cancer Center, Baylor University Medical Center, Dallas, Texas, United States of America; 5 Department of Pathology, Erasmus MC University Medical Center, Rotterdam, the Netherlands; 6 Department of Pathology, Radboud UMC, Nijmegen, the Netherlands; 7 Department of Genetics, Center for Molecular Medicine, University Medical Center Utrecht, Utrecht, The Netherlands; Sapporo Ika Daigaku, JAPAN

## Abstract

**Objective:**

Overall and splice specific expression of Spleen Tyrosine Kinase (*SYK*) has been posed as a marker predicting both poor and favorable outcome in various epithelial malignancies. However, its role in colorectal cancer is largely unknown. The aim of this study was to explore the prognostic role of *SYK* in three cohorts of colon cancer patients.

**Methods:**

Total messenger RNA (mRNA) expression of *SYK*, *SYK(T)*, and mRNA expression of its two splice variants *SYK* short *(S)* and *SYK* long *(L)* were measured using quantitative reverse transcriptase (RT-qPCR) in 240 primary colon cancer patients (n = 160 patients with chemonaive lymph node negative [LNN] and n = 80 patients with adjuvant treated lymph node positive [LNP] colon cancer) and related to microsatellite instability (MSI), known colorectal cancer mutations, and disease-free (DFS), hepatic metastasis-free (HFS) and overall survival (OS). Two independent cohorts of patients with respectively 48 and 118 chemonaive LNN colon cancer were used for validation.

**Results:**

Expression of *SYK* and its splice variants was significantly lower in tumors with MSI, and in *KRAS* wild type, *BRAF* mutant and *PTEN* mutant tumors. In a multivariate Cox regression analysis, as a continuous variable, increasing *SYK(S)* mRNA expression was associated with worse HFS (Hazard Ratio[HR] = 1.83; 95% Confidence Interval[CI] = 1.08–3.12; p = 0.026) in the LNN group, indicating a prognostic role for *SYK(S)* mRNA in patients with chemonaive LNN colon cancer. However, only a non-significant trend between *SYK(S)* and HFS in one of the two validation cohorts was observed (HR = 4.68; 95%CI = 0.75–29.15; p = 0.098).

**Conclusion:**

In our cohort, we discovered *SYK(S)* as a significant prognostic marker for HFS for patients with untreated LNN colon cancer. This association could however not be confirmed in two independent smaller cohorts, suggesting that further extensive validation is needed to confirm the prognostic value of *SYK(S)* expression in chemonaive LNN colon cancer.

## Introduction

Colon cancer is the second most common malignancy in the Western World with close to 450,000 new cases in Europe in 2012 [1]. As in most solid cancers, histological tumor staging (TNM) is the best determinant of prognosis and as a result provides recommendations for treatment decisions. The current treatment for stage I-III colon cancer is surgery alone for stages I and II, and surgery combined with adjuvant chemotherapy for stage III. However, up to 21% of the patients with stage I-II and up to 40% of the patients with stage III colon cancer will develop metastatic disease after curative surgery [2, 3]. Therefore, prognostic biomarkers complementing the TNM classification are urgently needed [4, 5].

Tyrosine-protein kinases are key regulators of cell proliferation associated with poor survival and tumorigenesis, and are therefore extensively studied in the field of oncological biomarker research [6, 7]. Spleen tyrosine kinase (SYK) has been posed as marker predicting both poor and favorable outcome in various epithelial malignancies including colorectal cancer [8–11]. However, most of these studies have focused on functional outcome in cell lines or associated tumor characteristics to the total mRNA or protein expression of SYK instead of linking mRNA and/or protein expression of SYK to long term clinical outcome. Furthermore, evidence suggesting different biological effects for the two known splice variants of SYK on growth properties of cancer cells is accumulating. In aggregate, the long isoform SYK(L) appears to be associated with tumor suppressive activities while the short isoform SYK(S) appears to be associated with tumor promoting activities. For instance, in patients with hepatocellular cancer, the expression of SYK(S) has been reported to be a significant indicator of poor prognosis [12].

The significance of SYK and its isoforms in colorectal cancer is largely unknown. Yang et al. showed that hypermethylation of the *SYK* promoter region resulted in loss of overall *SYK* mRNA expression, which was associated with a higher tumor stage and reduced five-year overall survival in a heterogeneous group of stage I-IV colon and rectum carcinoma [13]. In a second study by Ni et al. SYK(L) but not SYK(S) was downregulated in the majority of cancer and adjacent non-cancerous colon tissues [14]. Lastly, *SYK* is part of various prognostic gene signatures and the gene set used to define the consensus molecular subtypes of colorectal cancer [15, 16].

We aimed to assess the association of mRNA expression of overall *SYK* (*SYK(T)*) and its splice variants *SYK(L)* and *SYK(S)* with disease outcome in a well-defined homogeneous prospectively collected set of primary tumor tissues of patients with stage I-III colon cancer. Patients with lymph node negative (LNN) colon cancer who did not receive systemic adjuvant chemotherapy (chemonaive) and patients with lymph node positive (LNP) colon cancer who did receive adjuvant chemotherapy were analyzed separately to distinguish between pure disease prognosis and prognosis after adjuvant chemotherapy.

## Material and methods

Where possible, the guidelines for Reporting recommendations for tumour MARKer (REMARK) prognostic studies were followed, and the paper was written accordingly [17].

### Patient selection

Patients were selected from the MATCH study, an ongoing prospective multicenter observational cohort study from 2007 onwards including adult patients who undergo curative surgery in one of seven participating hospitals in the Rotterdam region, the Netherlands. Patients received treatment according to the current national guideline [18]. Patients were verbally informed about the storage and use of tissue samples, and the collection of clinical data for research purposes. The institutional review board of the Erasmus MC University Medical Center approved the MATCH study and specifically approved studies on (epi)genetic biomarkers to predict recurrence of diseases including the current study (Institutional Review Board number MEC 2007–088). Written informed consent was obtained from all patients.

**Fig 1:**
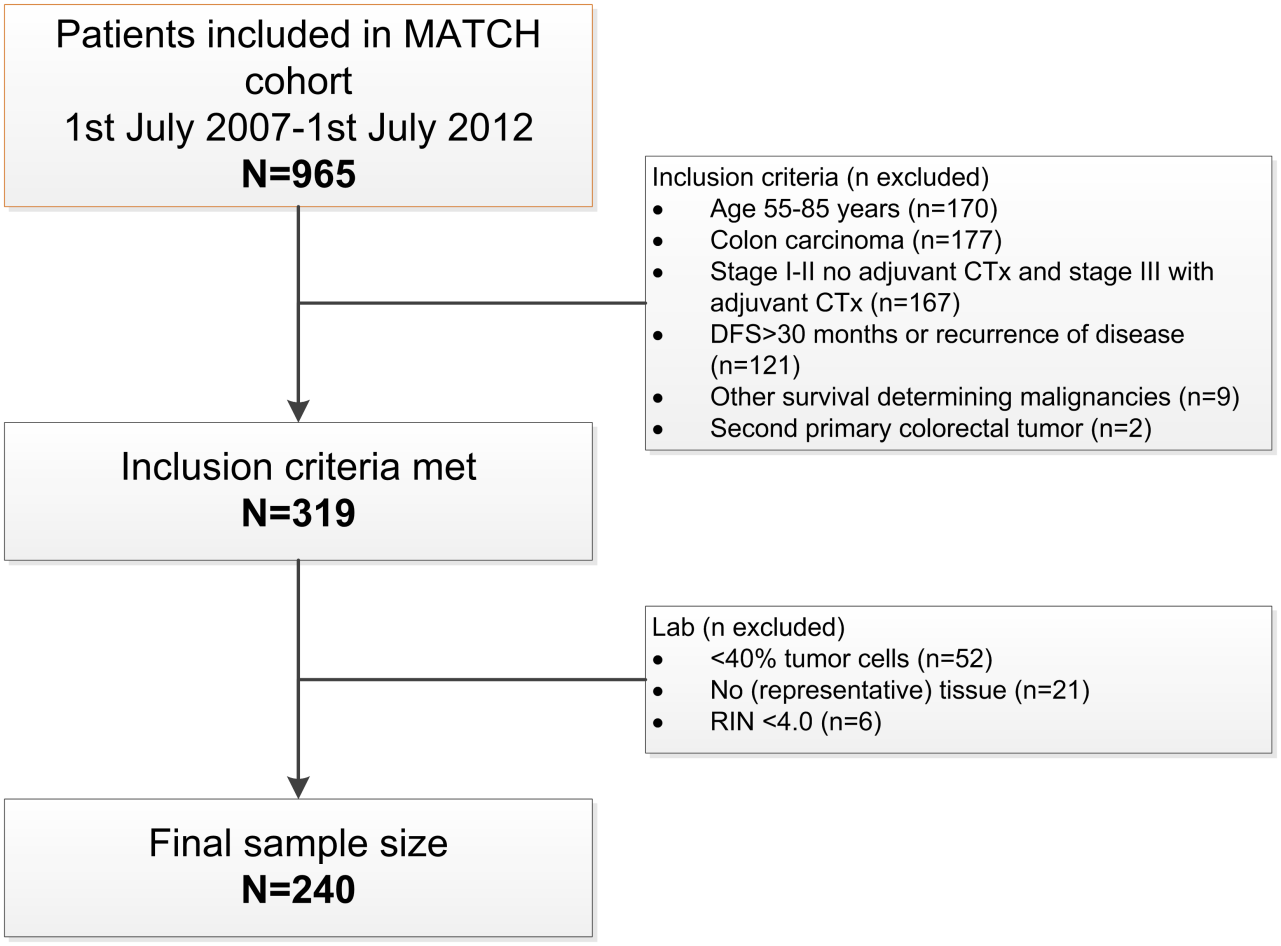
Diagram of analysis workflow of the MATCH cohort.

Inclusion criteria for this study were: informed consent available, inclusion date between 1^st^ July 2007 and 1^st^ July 2012 to ensure sufficient follow up, age > 55 years, stage I-II without adjuvant chemotherapy or stage III with adjuvant chemotherapy, radical surgery, fresh frozen tissue with at least 40% invasive tumor cells available, and either recurrence of disease or at least 30 months of disease-free follow-up. A diagram of the analysis workflow is shown in Fig 1.

The two independent validation cohorts consisted of 84 and 196 fresh frozen samples of primary colorectal cancers obtained through the Baylor Scott and White Research Institute and Charles A Sammons Cancer Center (Dallas, TX, USA) (cohort A and cohort B). Details on samples collection, processing and RNA isolation have been described previously [19]. For 82 and 185 patients of these cohorts respectively, RNA was sent to our lab to perform the cDNA synthesis and mRNA transcript level quantifications using the methodology as was used for the discovery study (see below). In cohort A, 34 patients were excluded (failed RNA/cDNA quality control [n = 10], rectal carcinoma [n = 23] and irradical resection [n = 1]) leaving a total of 48 patients for analysis (S1 Fig). In cohort B, 80 patients were excluded (failed RNA/cDNA quality control [n = 7], rectal carcinoma [n = 51] and age < 50 years [n = 9] and incomplete survival data [n = 2]) leaving a cohort of 116 patients for analysis (S2 Fig).

### Sample collection and processing

Immediately following removal of the resection specimen during surgery, the specimen was transported to the pathology lab at room temperature and without any conservation fluids. In the pathology lab, two to four biopsies of both central and peripheral regions of the tumor as well as one or two adjacent non-tumor colon tissue samples were taken and fresh frozen with a maximum cold ischemia time of two hours. All samples were stored in liquid nitrogen.

### RNA isolation, cDNA synthesis and mRNA transcript level quantification

Sectioning of fresh frozen colon cancer and normal colon tissue was done using a cryostat microtome (Thermo Scientific Microm HM 560, Thermo Fisher Scientific, inc.) set at -20°C. Before, during and after sectioning for RNA isolation, 3 x 5 μm sections were cut and after hematoxylin-eosin (HE) staining reviewed by two pathologists independently. For the MATCH cohort, the percentage of tumor cells, necrosis, infiltrate and normal cells were estimated relative to other cells (eg, stromal cells, inflammatory infiltrate and pre-existing epithelial cells). The estimates were scored in categories of 0–5%, 6–10%, 11–20%, 21–30%, 31–40%, 41–50%, 51–60%, 61–70%, 71–80%, 81–90%, and 91–100% tumor cells. Differentiation grade of the tumor was estimated according to the WHO 2010 classification for the carcinoma of the colon and rectum (WHO Press, World Health Organization, 20 Avenue Appia, Geneva, Switzerland). For the validation cohorts, no HE slides were available for evaluation.

For the discovery cohort, RNA was isolated from 30 μm sections using RNA-Bee^®^ according to the manufacturer’s instructions (Tel-Test inc., USA). For the validation cohorts, RNA was isolated with the RNeasy tissue kit (Qiagen, Germany). Quality and quantity of RNA was assessed with the Nanodrop ND-1000 (Thermo Scientific, Wilmington, USA) and the MultiNA Microchip Electrophoresis system (Shimadzu, Kyoto, Japan). Next, cDNA was generated from 2 μg of the isolated total RNA for the discovery cohort and from 0.1–1 μg of the isolated total RNA for the validation cohort using Reverse Transcriptase (RT) with the Thermo Scientific RevertAid H Minus First Strand cDNA Synthesis Kit (Fermentas, Thermo Scientific, USA) using the protocol supplied by the manufacturer, followed by an RNAse H step (Ambion, Life Technologies, USA) to digest any remaining RNA. Quantitative real-time PCR (qPCR) was performed with the Mx3000P QPCR machine (Agilent Technologies, NL) using ABgene Absolute Universal or Absolute SYBR Green with ROX PCR reaction mixtures (Thermo Scientific, USA) according the manufacturer’s instructions [20].

*SYK* mRNA expression levels were quantified with commercially available and validated TaqMan assays (Applied Biosystems, Thermo Scientific, USA) for the total expression of *SYK* (*SYK(T)*); *Hs00374292_m1*, and for its two alternative splicing variants, full-length *SYK* (*SYK(L)*); *Hs00895384_m1* and the short gene product lacking a 23-amino acid insert within the "linker" region located between the second Src homology 2 and the catalytic domain (*SYK(S)*); *Hs00177369_m1*. *SYK* mRNA expression levels were normalized using the average of three reference genes (*HMBS*, *HPRT1* and *TBP*) using the 2-ΔΔCq method as described in detail before by Livak and Schmittgen [21] and Sieuwerts et al [22], using a serially diluted pooled tumor cDNA sample as calibrator in every run to allow comparisons between runs. Only cDNA samples that were at a 100-fold final dilution in the qPCR able to generate a Cq value for the average of the reference genes within 28 cycles were considered of sufficient quality and quantity to be included in the study. Specifics of the gene assays used are provided in S1 Table.

### Mesenchymal and infiltrate markers

To capture epithelial to mesenchymal transition (EMT), the mRNA expression levels of one epithelial marker (*EPCAM*) and the three mesenchymal markers from the Oncotype Dx (*BGN*, *FAP*, *INHBA*) were measured using RT-qPCR [23].

*PTPRC* mRNA levels (a measure for CD45), which is present on all differentiated hematopoietic cells except erythrocytes and plasma cells, were used to estimate the contribution of infiltrate. Specifics of the gene assays to generate these indices are provided in S1 Table.

### Mutation calling

For n = 238 patients, RNA sequencing data was available [24]. In short, somatic genetic variations were detected in RNA-seq data using the GATK RNA-seq variant calling tool [25]. From the variant call list produced by the GATK workflow, we only retained calls that overlapped known cancer mutations present in the COSMIC database [26].

### Microsatellite instability (MSI)

MSI was analyzed with the MSI Analysis System from Promega, a fluorescent PCR-based assay for the detection of MSI in 5 mononucleotide repeat markers (BAT-25, BAT-26, NR-21, NR-24 and MONO-27) and two pentanucleotide repeat markers (Penta C and Penta D). The mononucleotide markers were used for MSI determination, and the pentanucleotide markers to detect potential sample mix ups and/or contamination using the protocol supplied by the manufacturer. In brief, genomic DNA was extracted with the NucleoSpin Tissue kit (Macherey-Nagel, BIOKE, Leiden, NL) from 2 to 5 x 30 μm sections cut in between the sections used for the RNA isolation. Quality and quantity were assessed by both Nanodrop, the Quant-iT PicoGreen dsDNA kit (Life Technnologies) and agarose gel electrophoresis. Next, 2 ng of PicoGreen measured DNA was used in the analysis for MSI.

The technical personnel performed all the above-mentioned analyses blinded from clinical outcome since they received the samples with according sample numbers and had no access to the patient identifying data nor the clinical data.

### Survival data

Disease free survival (DFS) was defined as the time elapsed between the date of surgery, and either the date of any recurrence of disease or the date of the last follow-up visit at which a patient was considered to have no recurrence.

Hepatic metastasis free survival (HFS) was defined as the time elapsed between the date of surgery, and either the date of the appearance of liver metastasis or the date of the last follow-up visit at which a patient was considered to have no liver metastases.

Overall survival (OS) was defined as the time elapsed between the date of surgery, and either the date of death or the date of the last check in the Municipal Personal Records Database.

### Statistical analyses

Statistical analyses were performed using the SPSS statistical package version 21. mRNA expression levels of *SYK(T)*, *SYK(S)* and *SYK(L)* were correlated with each other, the epithelial, mesenchymal and infiltrate markers, the clinicopathological characteristics and assessed CRC mutations using the Spearman Rank correlation test, Mann-Whitney U test, Kruskal-Wallis test and Jonckheere-Terpstra test where appropriate. Univariate Cox regression analysis was used to assess the association of the mRNA expression levels of *SYK(T)*, *SYK(S)* and *SYK(L)* as a continuous variable and clinicopathological characteristics with the clinical endpoints. Kaplan Meier estimates were used to visualize the association between mRNA expression of SYK and its splice variants with the relevant clinical endpoints. To this end, mRNA expression levels were split at the median level. Multivariate Cox regression analysis was used to assess the association between mRNA expression and clinical outcome while correcting for other clinicopathological factors associated with the clinical endpoint of interest. All analyses were two-sided and P<0.05 was considered significant.

## Results

### Correlation of mRNA expression levels *SYK(T)* and its splice variants

First, we assessed the correlation between *SYK(T)*, *SYK(S)* and *SYK(L)*. *SYK(T)* showed a good correlation with both *SYK(S)* and *SYK(L)* (Spearman’s Rho (r_s_) = 0.74, p<0.001 and r_s_ = 0.86 p<0.001, respectively) while *SYK(S)* and *SYK(L)* expression levels showed only a moderate association (r_s_ = 0.48 p<0.001) (S3 Fig). The worse correlation between the two splice variants suggested that a separate analysis of the splice variants may be of added value.

### Association of *SYK* mRNA expression levels with clinical and histopathological characteristics

In total, 240 patients were included in the discovery cohort. Clinical and histopathological characteristics, and median *SYK(T)*, *SYK(S)* and *SYK(L)* mRNA expression levels and their associations for the entire group are shown in Table 1, for the 160 patients with lymph node negative (LNN) disease in Table a in S2 Table and for the 80 patients with lymph node positive (LNP) disease in Table b in S2 Table.

**Table 1:** Clinical and histopathological characteristics of the total MATCH cohort.

		n	%	*SYK(T)* *median (IQR)*	*P value*	*SYK(S)* *median (IQR)*	*P value*	*SYK(L)* *median (IQR)*	*P value*	*Performed test*
*Gender*	Female	112	46.7%	-4.24 (-4.60 • -3.72)	0.11	-4.81 (-5.47 • -4.14)	0.18	-4.76 (-5.16 • -4.17)	0.40	*Mann-Whitney U*
	Male	128	53.3%	-4.05 (-4.58 • -3.50)		-4.63 (-5.27 • -3.93)		-4.66 (-5.19 • -4.03)		
*Age*		240	100%	-0.08	0.21	-0.009	0.89	-0.011	0.86	*Spearman's Rho*
*Tumor stage*	Stage I	60	25.0%	-4.31 (-4.71 • -3.67)	0.31	-4.62 (-5.21 • -3.96)	0.037	-4.73 (-5.26 • -4.28)	0.45	*Jonckheere-Terpstra*
	Stage II	100	41.7%	-3.96 (-4.55 • -3.47)		-4.61 (-5.36 • -4.07)		-4.54 (-5.04 • -3.95)		
	Stage III	80	33.3%	-4.15 (-4.58 • -3.68)		-4.98 (-5.70 • -4.19)		-4.81 (-5.30 • -4.33)		
*T status*	T2	71	29.6%	-4.32 (-4.69 • -3.76)	0.03	-4.69 (-5.26 • -3.97)	0.37	-4.77 (-5.33 • -4.46)	0.021	*Mann-Whitney U*
	T3	169	70.4%	-4.05 (-4.57 • -3.56)		-4.70 (-5.43 • -4.08)		-4.65 (-5.11 • -4.04)		
*Nodal status*	N0 ≥ 10 nodes assessed	131	54.6%	-4.08 (-4.60 • -3.62)	0.97	-4.63 (-5.32 • -4.08)	0.07	-4.62 (-5.13 • -4.04)	0.037	*Jonckheere-Terpstra*
	N0 < 10 nodes assessed	29	12.1%	-3.81 (-4.66 • -3.43)		-4.30 (-5.28 • -3.74)		4.68 (-5.17 • -3.90)		
	N1	53	22.1%	-4.09 (-4.48 • -3.64)		-4.89 (-5.68 • -4.21)		-4.77 (-5.34 • -4.38)		
	N2	27	11.3%	-4.30 (-4.60 • -3.69)		-5.08 (-5.74 • -4.03)		-4.96 (-5.21 • -4.31)		
*Tumor grade*	Good	20	8.3%	-3.95 (-4.43 • -3.46)	0.57	-4.61 (-5.30 • -3.88)	0.28	-4.59 (-4.81 • -3.97)	0.61	*Jonckheere-Terpstra*
	Moderate	192	80.0%	-4.14 (-4.60 • -3.61)		-4.69 (-5.33 • -4.08)		-4.72 (-5.20 • -4.15)		
	Poor	20	8.3%	-4.10 (-4.60 • -3.74)		-4.95 (-5.87 • -4.35)		-4.78 (-5.24 • -4.08)		
	Other	8	3.3%	-3.94 (-4.62 • -3.53)		-4.30 (-5.79 • -3.79)		-4.48 (-4.86 • -4.03)		
*Location*	Right	121	50.4%	-4.24 (-4.71 • -3.70)	0.015	-4.89 (-5.58 • -4.15)	0.004	-4.84 (-5.28 • -4.28)	0.008	*Mann-Whitney U*
	Left	119	49.6%	-4.01 (-4.47 • -3.53)		-4.52 (-5.21 • -3.85)		-4.62 (-4.96 • -4.13)		
*MSI status*^a^	MSI	49	20.4%	-4.59 (-5.02 • -4.25)	<0.001	-5.34 (-5.77 • -4.86)	<0.001	-5.05 (-5.49 • -4.54)	<0.001	*Mann-Whitney U*
	MSS	190	79.2%	-3.96 (-4.47 • -3.49)		-4.46 (-5.21 • -3.92)		-4.63 (-5.08 • -4.03)		

*SYK* mRNA expression levels were normalized using the average of three reference genes (*HMBS*, *HPRT1* and *TBP*) using the 2-ΔΔCq method as described in detail before by Livak and Schmittgen [21] and Sieuwerts et al [22].

^a^ n = 1 missing

A significantly lower expression of *SYK(T)*, *SYK(S)* and *SYK(L)* was found in MSI tumors as compared to MicroSatellite Stable (MSS) tumors. This finding was observed in the total group as well as in both subgroups, except for *SYK(L)* in the LNP group. *SYK* expression was also significantly associated with tumor stage and location, but significance was dependent on the type of variant analyzed. While expression of *SYK(S)* was higher in stage I and II than in stage III, expression of *SYK(T)* and *SYK(L)* was not found to correlate in an unambiguous way with tumor stage. Independent of stage, a higher expression of *SYK(T)*, *SYK(S)* and *SYK(L)* was found in left sided tumors, which was also observed for *SYK(S)* in the LNN group and for *SYK(T)* and *SYK(L)* in the LNP group.

These data indicated a differential expression of *SYK* splice variants as compared to total *SYK* expression, with significant differences in mRNA expression of *SYK(T)*, *SYK(S)* and/or *SYK(L)* with MSI status, stage and tumor location.

### Association of *SYK* mRNA expression levels and mesenchymal markers

To explore the association between the *SYK* isoform variants and features of EMT in our cohort, mRNA expression levels of one epithelial marker (*EPCAM*) and the three mesenchymal markers from the Oncotype Dx [23] (*BGN*, *FAP*, *INHBA*) were measured using RT-qPCR (S3 Table). mRNA expression levels of *SYK(T)*, *SYK(S)* or *SYK(L)* all showed a moderate positive correlation with mRNA expression of *EPCAM* (r_s_ = 0.47 p<0.001, r_s_ = 0.58 p = 0.001 and r_s_ = 0.41 p<0.001, respectively). For the stromal markers, only *FAP* showed a significant but less striking negative association with *SYK(S)* in the total group (r_s_ = -0.13 p = 0.046) and LNP group (r_s_ = -0.24 p = 0.031).

### Association of *SYK* mRNA expression levels and infiltrate

As *SYK* is a known infiltrate marker [27], we next explored the association between mRNA and protein expression levels of *SYK* and its isoform variants, and the extent of possible infiltrate contribution. We measured mRNA expression levels of an infiltrate marker (*PTPRC*/CD45) using RT-qPCR and scored the percentage of infiltrate on H&E slides. Although mRNA expression levels of *SYK(S)* correlated moderately negatively with the percentage of infiltrate as scored by a pathologist in the total group (r_s_ = -0.14 p = 0.043), we did not observe a significant association between *PTPRC*/CD45 and mRNA expression of *SYK* or its splice variants (S4 Table).

### Association of *SYK* mRNA expression levels with known CRC mutations

**Fig 2:**
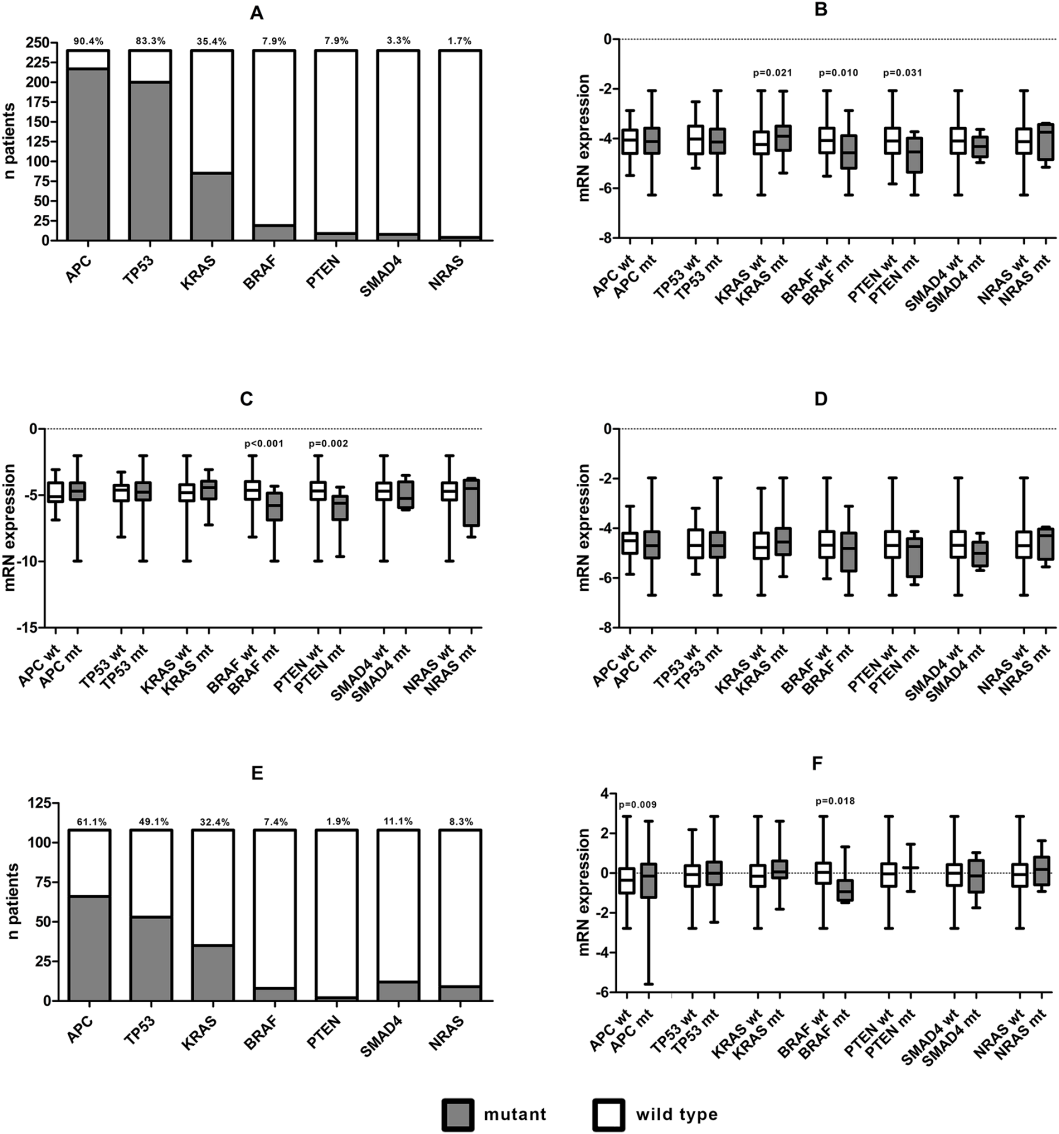
The association between SYK mRNA expression and known CRC mutations. Mutation rates in the MATCH cohort (n = 240)(a); differences in mRNA expression of *SYK(T)* (b), *SYK(S)* (c) and *SYK(L)* in the MATCH cohort; mutation rates in the TCGA (n = 108)(e); differences in mRNA expression of *SYK(T)* in the TCGA cohort (f).

Because of the correlation of *SYK* mRNA expression with MSI and a previous study which showed that *SYK* is differentially expressed in *KRAS*-dependent and *KRAS*-independent cancer cell lines [28], we explored the association between known CRC mutations and *SYK* expression in our MATCH cohort and TCGA (Fig 2). The mutation rates were: *APC* 90.4%, *TP53* 83.3%, *KRAS* 35.4%, *BRAF* 7.9%, *PTEN* 3.8%, *SMAD4* 3.3% and *NRAS* 1.7% (Fig 2a). mRNA expression of *SYK(T)* was significantly higher in *KRAS* mutant (mt), and lower in *BRAF* mt and *PTEN* mt tumors compared to wild type (wt) tumors (p = 0.021, p = 0.01 and p = 0.031, respectively) (Fig 2b). A similar association was observed for *SYK(S)* (*BRAF* p<0.001 and *PTEN* p = 0.002, respectively), while no significant associations were found for *SYK(L)* (Fig 2c and 2d). In line with literature [29], these mutations were more prevalent in MSI tumors than in MSS tumors (*BRAF* 30.6% vs 2.1%, p<0.001 and *PTEN* 10.2% vs 2.1% p = 0.008). No significant differences in mRNA expression for *SYK(T)*, *SYK(S)* and *SYK(L)* were observed.

Next, we analyzed all cases of stage I-III colon cancer in the TCGA for which both the known CRC mutations and *SYK(T)* expression levels were available (n = 108) (Fig 2e). In this cohort, *SYK(T)* expression was significantly lower in *BRAF* mt tumors compared to wild type tumors (p = 0.0018) and significantly lower in *APC* mt tumors compared to wild type tumors (p = 0.009) (Fig 2f).

### Association of *SYK* mRNA expression levels with survival

First, associations between basic patient characteristics and survival outcome were assessed using Cox regression analysis. In the total MATCH cohort, having a stage III tumor or more than three positive lymph nodes (N2 versus N0) was significantly associated with an adverse DFS. Age, gender and more than three positive lymph nodes were significantly associated with poor OS (Table a in S5 Table). In the LNN subgroup, less than ten lymph nodes assessed in total was associated with an adverse HFS. In this sub group, only age at time of surgery was significantly associated with OS (Table b in S5 Table). In the LNP group, more than three positive lymph nodes was significantly associated with an adverse DFS. Also, the presence of more than three positive lymph nodes and increasing age were significantly associated with poor OS in the LNP subgroup of the MATCH cohort (Table c in S5 Table).

Subsequently, the associations between mRNA expression levels of *SYK* and its splice variants with DFS, HFS and OS were assessed using Cox regression analysis. For the whole MATCH cohort (n = 240), no significant associations were found between mRNA expression of *SYK(T)*, *SYK(S)* and *SYK(L)*, and the clinical endpoints (Table a in S5 Table). Next, the LNN chemonaive group (n = 160) and the LNP group who had received adjuvant therapy (n = 80) were analyzed separately.

**Fig 3:**
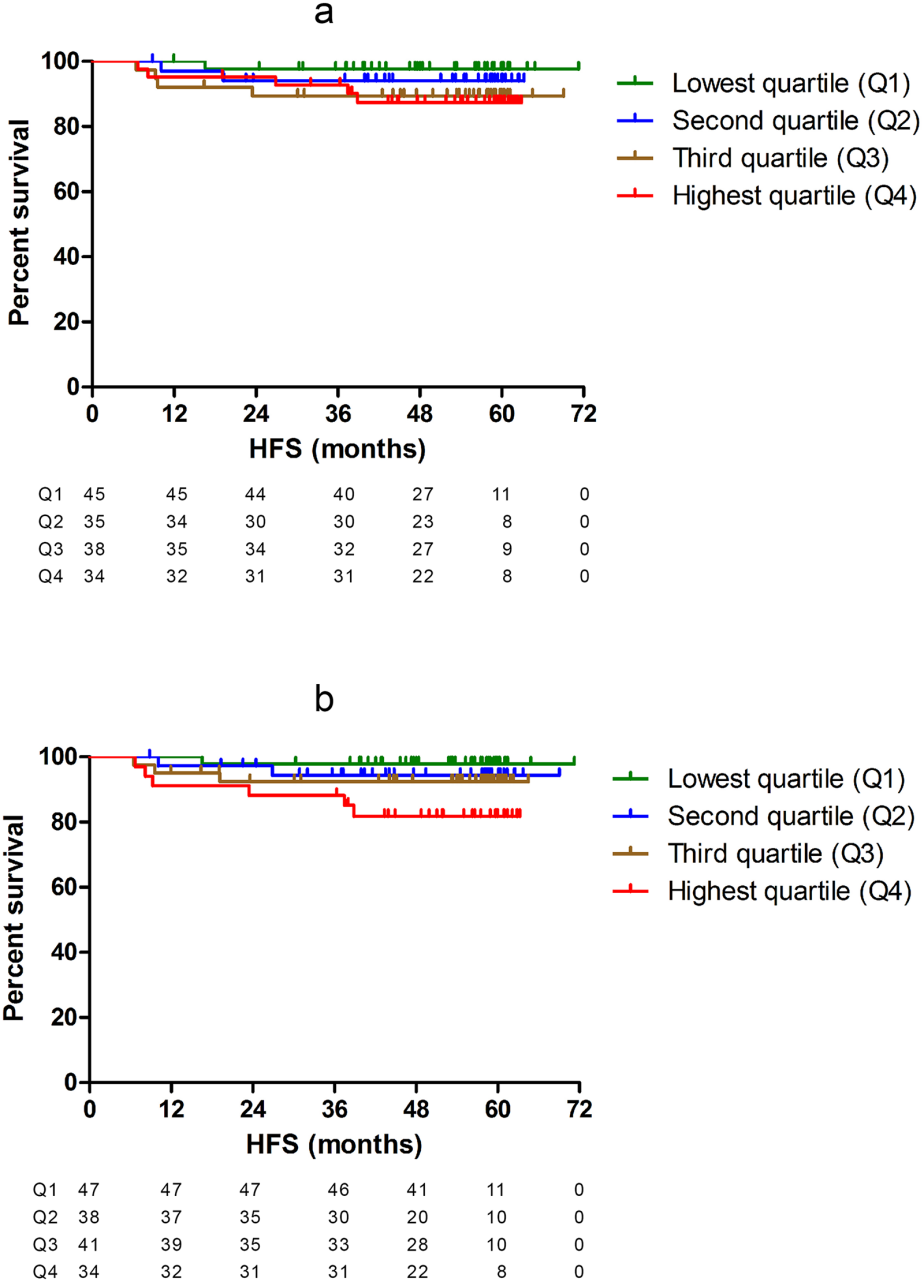
Survival curves for HFS in the LNN subgroup of the MATCH cohort for *SYK(T)* split in quartiles (a) and for *SYK(S)* split in quartiles (b).

In the LNN group, higher mRNA expression levels of *SYK(T)* and *SYK(S)* (continuous variables) were significantly associated with poor HFS (Hazard Ratio [HR] = 2.05; 95% Confidence Interval [CI] = 1.01–4.17; p = 0.047 and HR = 2.14; 95% CI = 1.14–4.01; p = 0.018, respectively) (Table b in S5 Table). The association of mRNA expression of *SYK(T)* and *SYK(S)* split into four quartiles (Q1 with lowest mRNA expression levels through Q4 with the highest mRNA expression levels) with HFS was visualized by Kaplan-Meier curves (Fig 3a and 3b), which suggested an impaired HFS particularly for patients with *SYK(S)* mRNA expression levels of the tumor in Q4. These findings were confirmed in an exploratory analysis with Cox regression analysis showing a significantly worse HFS for Q4 versus Q1-Q3 (HR = 3.83; 95%CI = 1.23–11.86; p = 0.02). To explore the prognostic role of *SYK(S)* for HFS independent of other significantly associated factors in the LNN group, we performed a multivariate Cox regression model including N-status, the only other factor significantly related to HFS in the LNN group, and *SYK(S)* mRNA expression level. In this analysis, both continuous mRNA expression levels of *SYK(S)* and nodal status remained significantly associated with HFS (HR = 1.83; 95% CI = 1.08–3.12; p = 0.026 and HR = 1.27; 95%CI = 1.01–1.60; p = 0.042) (Table 2). However, since the total number of events in this low-risk group was only 12, these results should be interpreted with caution.

**Table 2:** Univariate and multivariate cox regression analysis for the LNN MATCH cohort.

		n	%	HFS (events = 12) HR (95%CI)	P value	HR (95%CI)	P value
*mRNA expression*	*SYK(S)*	160	100%	2.14 (1.14 • 4.01)	0.018	1.83 (1.08 • 3.12)	0.026
*Gender*	Female	78	48.8%	1			
	Male	82	51.3%	1.97 (0.59 • 6.53)	0.27		
*Age*		160	100%	1.01 (0.94 • 1.08)	0.79		
*Tumor stage*	Stage I	60	37.5%	1			
	Stage II	100	62.5%	1.21 (0.36 • 4.02)	0.76		
	Stage III	-					
*T status*	T2	60	37.5%	1			
	T3	100	62.5%	1.21 (0.36 • 4.02)	0.76		
*Nodal status*	N0 ≥ 10 nodes assessed	131	81.9%	1			
	N0 < 10 nodes assessed	29	18.1%	3.42 (1.09 • 10.78)	0.04	1.27 (1.01 • 1.60)	0.042
	N1	-					
	N2	-					
*Tumor grade*	Good	13	8.1%	1			
	Moderate	135	84.4%	0.88 (0.11 • 6.91)	0.27		
	Poor	9	5.6%	2.85 (0.26 • 31.39)	0.39		
	Other^a^	3	1.9%	-	-		
*Location*	Right	82	51.3%	1			
	Left	78	48.8%	1.09 (0.35 • 3.38)	0.88		
*MSI status*^b^	MSI	37	23.1%	1			
	MSS	122	76.1%	31.28 (0.12 • 8430.24)	0.23		

^a^ there were no events in this subgroup;

^b^ n = 1 missing

In the LNP group, no significant associations between any of the *SYK* mRNA expression levels and clinical endpoints were observed (Table c in S5 Table).

### Validation cohorts

Details on patient and tumor characteristics for both cohorts can be found in Table 3. More patients in cohort A and B had a T1/T4 tumor compared to patients in the LNN subgroup of the MATCH cohort (14.6% and 8.6% vs 0%, p<0.001 respectively). The total number of assessed lymph nodes was less often below the cut-off of 10 lymph nodes (cohort A 4.2% and cohort B 12.9% vs 18.1% in the LNN subgroup of the MATCH cohort, p<0.001). Both cohort A and B contained more well differentiated tumors compared to the LNN MATCH cohort (83.3% and 93.1% vs 8.1%, p<0.001 respectively). In cohort A, more tumors were right-sided compared to the LNN subgroup of the MATCH cohort (79.2% vs 51.3%, p>0.001). In cohort B, less tumors for which MSI status was known were MSI compared to the LNN subgroup of the MATCH cohort (9.5% vs 23.3%, p = 0.019). No differences for the distribution of gender, age, tumor stage, or location of recurrence between the validation cohorts and the LNN subgroup of the MATCH cohort were observed (Table 3). No significant association between mRNA expression of *SYK(T)* or its splice variants with any of these characteristics were observed.

**Table 3:** Basic characteristics of the LNN MATCH cohort, and validation cohorts A and B.

		MATCH cohort	Cohort A	Cohort B	P value
*Gender*	Female	78 (48.8%)	24 (50.0%)	51 (44.0%)	0.67
	Male	82 (51.3%)	24 (50.0%)	65 (56.0%)	
*Age*		68 (62–75)		69 (61–78)	0.89
*Tumor stage*	Stage I	60 (37.5%)	16 (33.3%)	37 (31.9%)	0.61
	Stage II	100 (62.5%)	32 (66.7%)	79 (68.1%)	
*T status*	T1	0 (0.0%)	3 (6.3%)	10 (8.6%)	<0.001
	T2	60 (37.5%)	13 (27.1%)	27 (23.3%)	
	T3	100 (62.5%)	28 (58.3%)	79 (68.1%)	
	T4	0 (0.0%)	4 (8.3%)	0 (0.0%)	
*Nodal status*	N0 ≥ 10 nodes assessed	131 (81.9%)	46 (95.8%)	101 (87.1%)	0.046
	N0 < 10 nodes assessed	29 (18.1%)	2 (4.2%)	15 (12.9%)	
*Tumor grade*	Good	13 (8.1%)	40 (83.3%)	108 (93.1%)	<0.001
	Moderate	135 (84.4%)	5 (10.4%)	0 (0.0%)	
	Poor	9 (5.6%)	2 (4.2%)	8 (6.9%)	
	Other	3 (1.9%)	1 (2.1%)	0 (0.0%)	
*Location*	Right	82 (51.3%)	38 (79.2%)	53 (45.7%)	<0.001
	Left	78 (48.8%)	10 (20.8%)	63 (54.3%)	
*MSI status*^a^	MSI	37 (23.1%)	-	6 (9.5%)	0.019
	MSS	122 (76.3%)	-	57 (90.5%)	
*Location of recurrence*	No recurrence	133 (83.1%)	41 (85.4%)	105 (90.5%)	0.65
	Local	2 (1.3%)	1 (2.1%)	2 (17%)	
	Hepatic	11 (6.9%)	2 (4.2%)	5 (4.3%)	
	Non hepatic	11 (6.9%)	3 (6.3%)	4 (3.4%)	
	Combined	3 (1.9%)	1 (2.1%)	0 (0.0%)	

^a^ n = 1 missing in the MATCH cohort and n = 53 missing in cohort B

In both cohorts, no significant associations were observed between mRNA expression of *SYK(T)* nor the splice variants with DFS or HFS, although a non-significant trend between mRNA expression levels *SYK(S)* and HFS was observed in cohort A (HR = 4.68; 95%CI = 0.75–29.15; p = 0.098).

## Discussion

In epithelial malignancies, both tumor promoting and tumor suppressing roles have been ascribed to *SYK*. Evidence suggesting different effects of the *SYK* splice variants on growth properties of cancer cells is accumulating [10]. The dual role of *SYK* in epithelial cancers combined with the scarce literature on the role of *SYK* and its splice variants in colorectal cancer provided a rationale to assess their prognostic value in primary tumors of colon cancer patients. This study showed that high mRNA expression level of *SYK(S)* is associated with short HFS in our MATCH cohort of chemonaive LNN colon cancer patients, although these findings could not be validated in two independent clinically less well-defined and smaller cohorts of patients with chemonaive LNN colon cancer.

Three major mechanisms through which *SYK* may affect cancer cell properties have been identified: *SYK* promoting cell survival through anti-apoptotic factors, *SYK* altering cellular differentiation programs regulating EMT and *SYK* altering cell motility. Importantly, SYK has two alternatively spliced variants, SYK(L) and SYK(S). In the short splice variant which a stretch of 23 amino acids in linker B (Exon 7) is spliced out. In normal hematopoietic cells, SYK(S) is intrinsically less active compared to SYK(L). In most epithelial cancers, overall *SYK* mRNA levels are higher in cancerous cells compared to normal cells of the same organ, including colon, suggesting a tumor promotor role of SYK in tumorigenesis [10].

However, *SYK* mRNA or SYK protein expression have been both positively and negatively associated with tumor characteristics such as tumor grade and tumor stage. This paradoxical association may be explained by the accumulating observations that SYK(S) and SYK(L) both have an active but opposing role in solid cancers [30, 31]. These opposing effects are generally attributed to a different location within the cell with SYK(L) being present in both the nucleus and cytoplasm, and SYK(S) being confined to the cytoplasm [12, 30, 32]. Wang and co-workers showed that SYK(L) was present in both normal and cancerous cells, and suppressed cell invasiveness in breast cancer cell lines. In contrast, SYK(S) was present only present in cancerous cells, but did not affect cell invasiveness [30]. Hong et al. observed similar differential expression patterns in hepatocellular carcinoma (HCC) as *SYK(L)* mRNA expression was downregulated in 38% of the tumor samples while *SYK(S)* mRNA expression was detectable in 40% of the tumor samples and none of the normal liver tissue samples. Furthermore, *SYK(S)* mRNA expression levels were higher in poorly differentiated tumors compared to well differentiated tumors, while *SYK(L)* was expressed vice versa [12]. Ni et al. showed that overexpression of SYK(L) significantly reduced cell proliferation *in vitro* while SYK(S) overexpression did not in the human colorectal cancer HCT116 cell line. They also observed downregulation of SYK(L) but not SYK(S) 69% of tumor tissue samples compared to adjacent non-cancerous tissues [14]. In the current study we observed an decreased mRNA expression of *SYK(S)* in stage III compared to stage I-II colon cancers, but no association between mRNA expression of the splice variants with tumor grade. The latter may be explained by the large portion (88.3%) of well to moderately differentiated tumors in our cohort. Overall, the findings in literature and the current study suggest that SYK(S) is associated with tumor promoting activities while SYK(L) is associated with tumor suppressing activities.

We also observed differential expression between left and right-sided tumors, MSI and MSS tumors, and between tumors with and without known CRC mutations. Right-sided tumors, MSI tumors, *BRAF* mt tumors and *PTEN* mt tumors expressed *SYK(T)* and *SYK(S)* at a significantly lower level compared to left-sided tumors, MSS tumors, and wild type tumors in both the total and LNN subgroup, respectively. The lower expression of *SYK(T)* and *SYK(S)* in tumors harboring a *PTEN* mutation supports the findings of a previous study on diffuse large B-cell lymphomas in which a subset of samples exhibited an increase in the *SYK* gene copy number variation while a different subset exhibited loss of *PTEN* suggesting two independent mechanisms to promote cell survival [33]. The association between high mRNA expression of *SYK(T)* and both splice variants and microsatellite stability is interesting, as microsatellite stability is considered to be a phenotype associated with poor prognosis [34]. In aggregate, these findings may suggest a different role for *SYK* in hypermutated versus non-hypermutated tumors, although these findings should be verified in independent cohorts. We also observed a higher expression of *SYK(T)* in *KRAS* mt compared to *KRAS* wt tumors in our own cohort. These findings were in line with a previous study reporting higher expression *KRAS*-dependent compared to *KRAS*-independent pancreatic and lung cancer cell lines [28]. Thus, *SYK* may play a different role in *KRAS* mt and *KRAS* wt tumors. Functional studies should be conducted in colorectal cancer cell lines and/or samples to confirm this assumption.

Next to the associations with tumor characteristics, we showed that high *SYK(S)* mRNA expression is associated with short HFS in our MATCH cohort of chemonaive LNN colon cancer patients. To our knowledge, one previous study of colorectal cancer patients explored the prognostic role of *SYK*. Yang et al. showed that methylation of the *SYK* gene promoter region was associated with decreased *SYK* mRNA and SYK protein expression, and subsequently showed a significantly worse five-year OS in the group with methylated *SYK* gene promoter region compared to the group with unmethylated *SYK* gene promoter region (5-year overall survival 59% vs 80% p<0.001, respectively [13]. However, the cohort consisted of stage I to IV colon and rectum carcinoma, and no details regarding neoadjuvant or adjuvant therapy and DFS were provided. Furthermore, only total expression of *SYK* was measured leaving questions regarding the prognostic value of the splice variants in their cohort unanswered. Interestingly, the prognostic role of the splice variants of *SYK* was investigated by Hong et al, who showed that patients with a *SYK(S)*-positive HCC were more likely to develop early and late recurrence (80.3% vs 53.8% P = 0.001 and 66.7% vs 16.7%; P = 0.002 respectively) compared to patients with a *SYK(S)*-negative HCC, which supports the findings in the MATCH cohort. Hong et al also showed that patients with a *SYK(S)*-positive HCC had a worse OS compared to patients with a *SYK(S)*-negative HCC [12]. We did not observe an association between *SYK* mRNA expression and OS in our cohort. Furthermore, we did not find evidence supporting a tumor suppressor role for *SYK(L)*.

Unfortunately, the findings in the MATCH cohort could not be confirmed in two independent cohorts of patients with chemonaive LNN colon cancer and therefore warrant further investigation. The different observations in the MATCH cohort and the two validation cohorts with regard to clinical outcome may be explained by the limited number of patients and events (especially in cohort A with 48 patients and only 3 events for HFS). Second, the observed differences may be explained by differences in tumor biology. The large majority of tumors in both validation cohorts were well-differentiated compared to a large majority of moderately differentiated tumors in the MATCH cohort. Furthermore, Cohort A contained significantly more right-sided tumors while cohort B contained significantly less MSI tumors compared to the LNN subgroup of the MATCH cohort. Beside the biological differences associated with these tumor characteristics, we showed that expression of *SYK(T)* and its splice variants was significantly different for left- vs right-sided tumors and for MSS vs MSI tumors in the MATCH cohort. Lastly, the two validation cohorts originated from Japan, which may account for some of the observed differences as worldwide variations in clinical outcome in colorectal cancer patients have been shown [35].

In conclusion, the differential expression of *SYK(T)* and its splice variants between left and right-sided tumors, MSI and MSS tumors, and tumors with and without a *BRAF* and/or *PTEN* mutation suggest a different role for *SYK* in hypermutated and non-hypermutated tumors. Furthermore, high *SYK(S)* was associated with poor HFS in the prospectively collected MATCH cohort of patients with chemonaive LNN colon cancer. However, the association was not confirmed in two independent, clinically less well-defined and smaller cohorts of patients with chemonaive LNN colon cancer. Further research is warranted to elucidate the role of *SYK* and its splice variants in colorectal cancer.

## Supporting information

S1 Fig Diagram of analysis workflow of validation cohort A. (TIF)

S2 Fig Diagram of analysis workflow of validation cohort B. (TIF)

S3 Fig Correlation plots and Pearson correlation coefficients of the correlation between *SYK(T)*, *SYK(S)* and *SYK(L)*. (TIF)

S1 Individual Patient Data Colon tumor sample overview, clinical characteristics and expression levels of SYK(T), SYK(S) and SYK(L). (XLSX)

S1 Table Gene assays used to measure mRNA expression of *SYK*, *SYK* splice variants and reference genes, and generate EMT, infiltrate and GGI indices. (PDF)

S2 Table Clinical and histopathological characteristics of the LNN subgroup (Table a) and the LNP subgroup (Table b) of the MATCH cohort. (PDF)

S3 Table The association between epithelial and mesenchyal markers and *SYK(T)*, *SYK(S) and SYK(L)* for the total MATCH chort, and the LNN and LNP subgroups of the MATCH cohort. (PDF)

S4 Table The association between infiltrate markers and *SYK(T)*, *SYK(S)* and *SYK(L)* for the total MATCH chort, and the LNN and LNP subgroups of the MATCH cohort. (PDF)

S5 Table Univariate cox regression analysis for the total MATCH cohort (Table a), and the LNN subgroup (Table b) and the LNP subgroup (Table c) of the MATCH cohort. (PDF)
