# Supplementary figures and images for: High mRNA expression of splice variant SYK short correlates with hepatic disease progression in chemonaive lymph node negative colon cancer patients
Source: PLoS One. 2017 Sep 28;12(9):e0185607. doi: 10.1371/journal.pone.0185607 (PMC5619807; doi:10.1371/journal.pone.0185607)

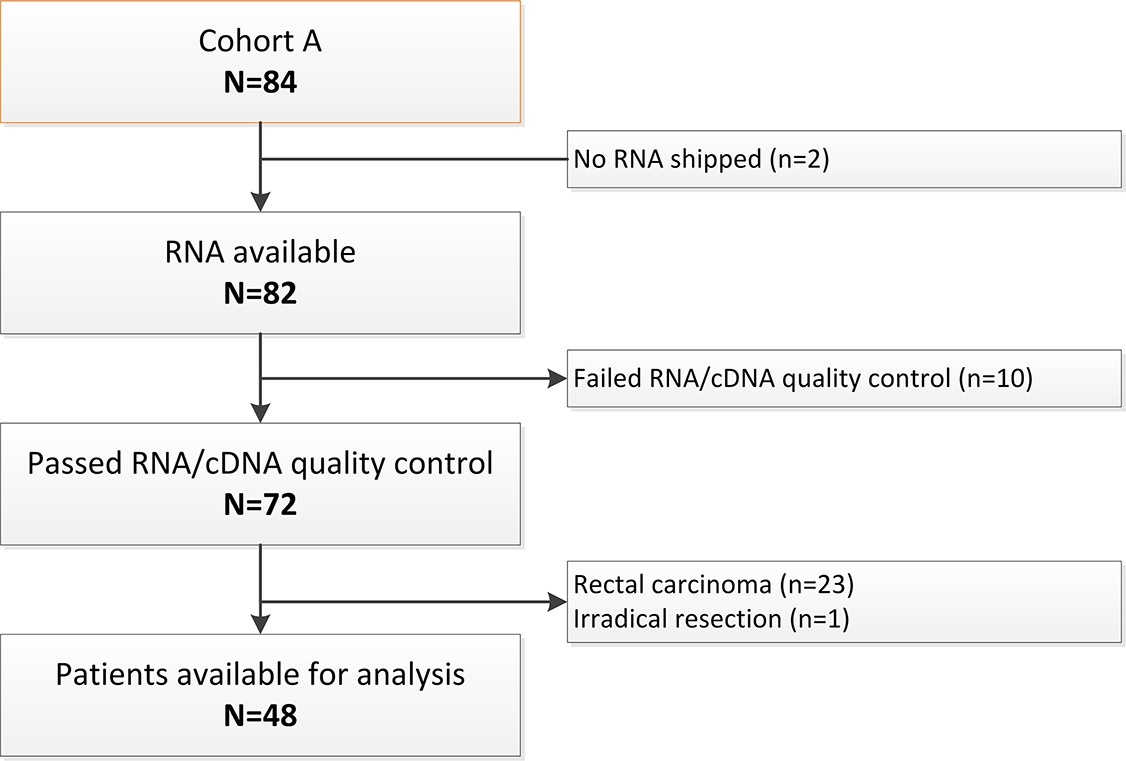

Supplement: S1 Fig — (TIF) [file pone.0185607.s001.tif]

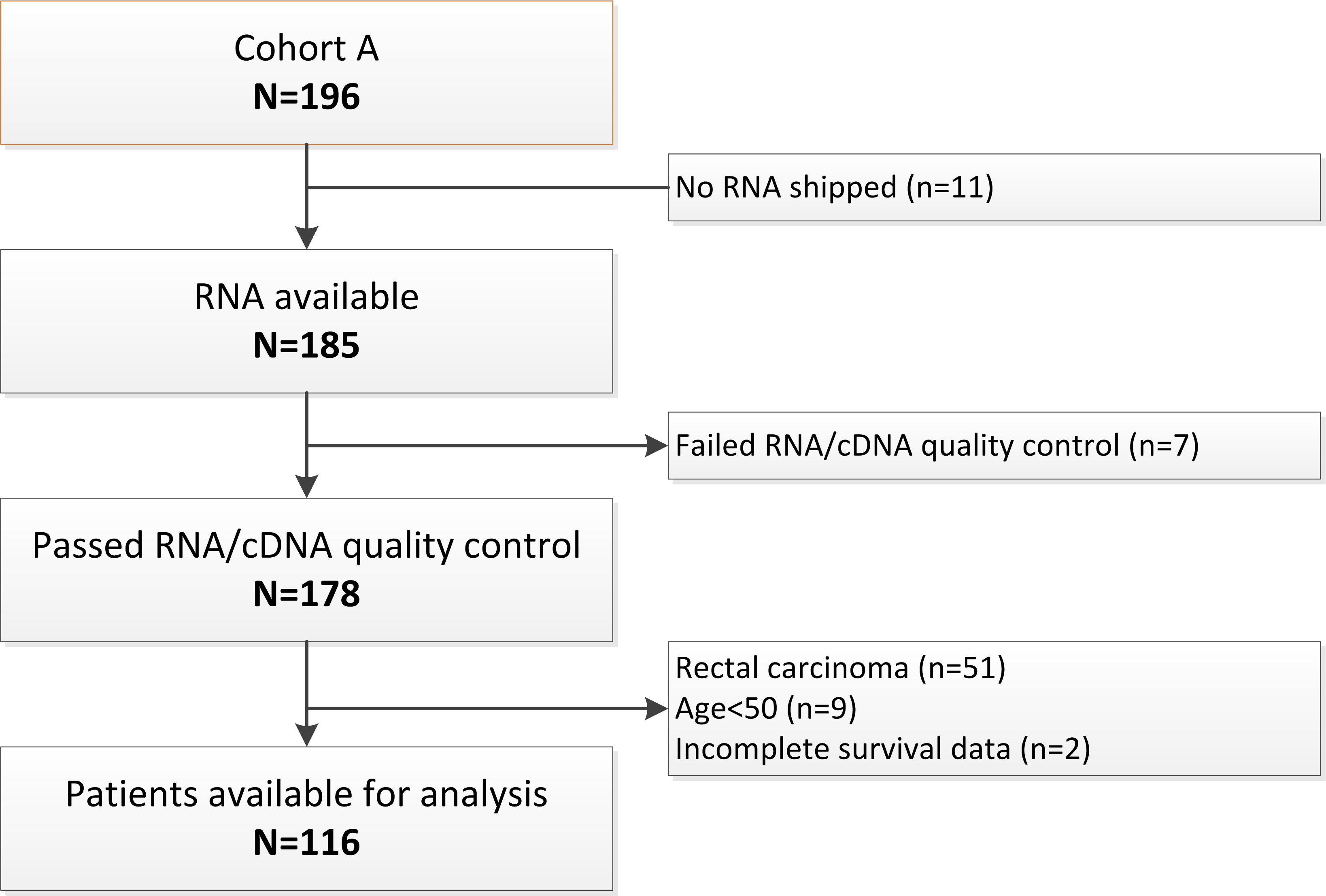

Supplement: S2 Fig — (TIF) [file pone.0185607.s002.tif]

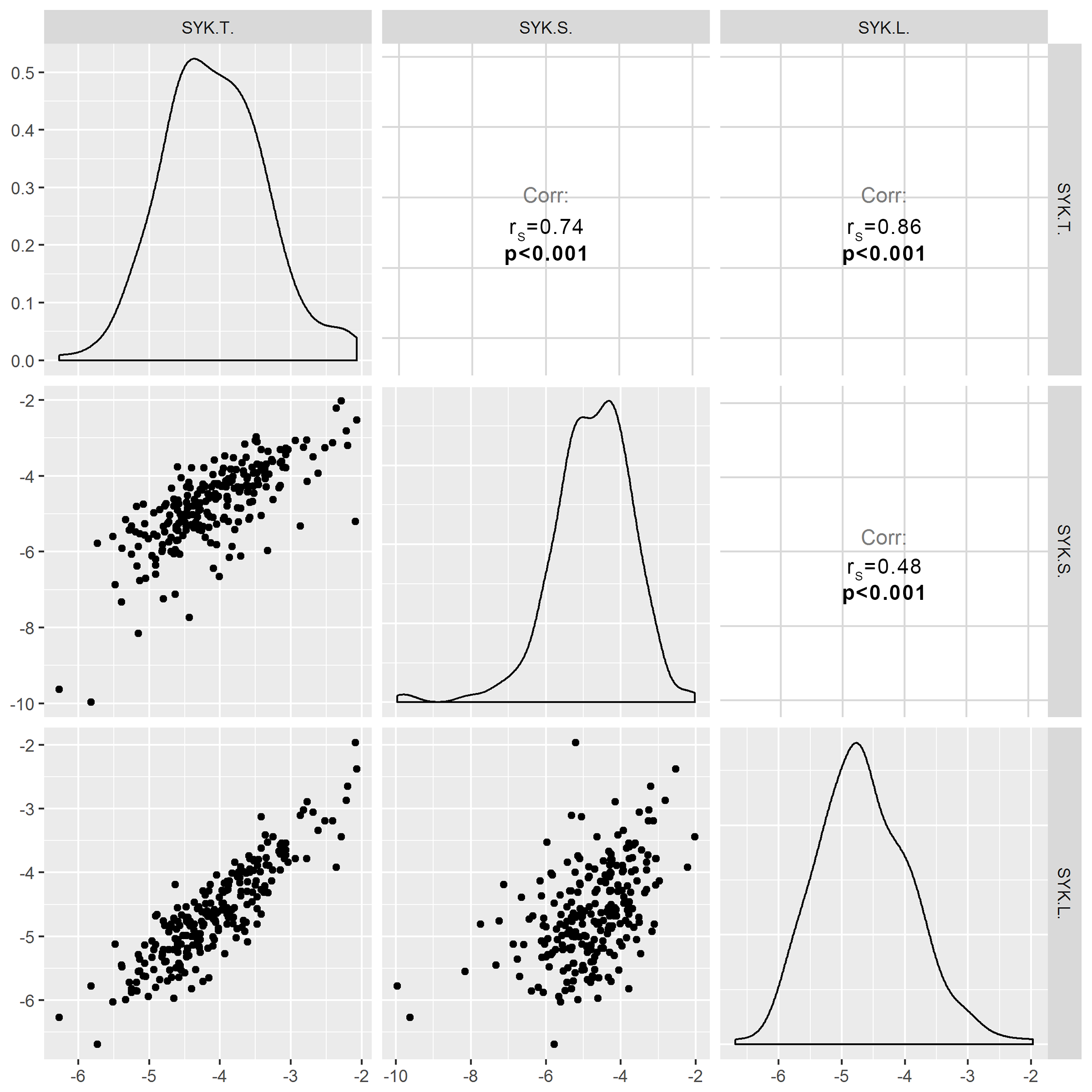

Supplement: S3 Fig — (TIF) [file pone.0185607.s003.tif]
