# Supplementary material for: High mRNA expression of splice variant SYK short correlates with hepatic disease progression in chemonaive lymph node negative colon cancer patients
Source: PLoS One. 2017 Sep 28;12(9):e0185607. doi: 10.1371/journal.pone.0185607 (PMC5619807; doi:10.1371/journal.pone.0185607)
Supplement: S1 Table — (PDF) [file pone.0185607.s005.pdf]

S1 Table. Gene assays used to measure mRNA expression of *SYK*, *SYK* splice variants and reference genes, and generate EMT, infiltrate and GGI indices.

INDEX	Gene Symbol	Gene Name	qPCR detection method	Assay ID ThermoFisher Scientific	F sequence	R sequence
SYK total	*SYK (T)*	Spleen Tyrosine Kinase	Taqman	Hs00374292_m1		
SYK short	*SYK (S)*	Spleen Tyrosine Kinase	Taqman	Hs00177369_m1		
SYK long	*SYK (L)*	Spleen Tyrosine Kinase	Taqman	Hs00895384_m1		
EMT	*BGN*	biglycan	Taqman	Hs00959141_g1		
EMT	*CDH2*	cadherin 2, type 1, N-cadherin (neuronal)	Taqman	Hs00983062_m1		
EMT	*FAP*	fibroblast activation protein, alpha	Taqman	Hs00990806_m1		
EMT	*FN1*	fibronectin 1	Taqman	Hs00277509_m1		
EMT	*INHBA*	inhibin, beta A	Taqman	Hs01081598_m1		
EMT	*EPCAM*	tumor-associated calcium signal transducer 1	SYBR		AGTTTGCGGACTGCACTTCA	AATACTCGTGATAAATTTTGGATCCA
EMT	*ESR1*	estrogen receptor 1	SYBR		ATCCTACCAGACCCTTCAGTG	GCCAGACGAGACCAATCATC
EMT	*ESR2*	estrogen receptor 2 (ER beta)	SYBR		CATGCTCCTGGCAACTACTTC	GCTCTTGGCAATCACCCAAAC
EMT	*IGF1*	insulin-like growth factor 1 (somatomedin C)	SYBR		TGGTGGATGCTCTTCAGTTC	GACAGAGCGAGCTGACTTG
EMT	*IGF2*	insulin-like growth factor 2 (somatomedin A)	SYBR		GCGGCTTCTACTTCAGCAG	CAGGTGTCATATTGGAAGAAC
EMT	*TGFB1*	transforming growth factor, beta 1	SYBR		GCCCTGGACACCAACTATTG	CGTGTCCAGGCTCCAAATG
EMT	*VIM*	vimentin	SYBR		CAGATTCAGGAACAGCATGTC	TCAGAGAGGTCAGCAAACTTG
EMT & Infiltrate	*VEGFA*	vascular endothelial growth factor A	Taqman	Hs00900055_m1		
Infiltrate	*PTPRC*	protein tyrosine phosphatase, receptor type, C, CD45	Taqman	Hs00236304_m1		
Reference gene	*HMBS*	hydroxymethylbilane synthase	SYBR		CATGTCTGGTAACGGCAATG	GTACGAGGCTTTCAATGTTG
Reference gene	*HPRT1*	hypoxanthine phosphoribosyltransferase 1	SYBR		TATTGTAATGACCAGTCAACAG	GGTCCTTTTCACCAGCAAG
Reference gene	*TBP*	TATA-box binding protein	SYBR		TTCGGAGAGTTCTGGGATTG	ACGAAGTGCAATGGTCTTTAG
