# Supplementary material for: High mRNA expression of splice variant SYK short correlates with hepatic disease progression in chemonaive lymph node negative colon cancer patients
Source: PLoS One. 2017 Sep 28;12(9):e0185607. doi: 10.1371/journal.pone.0185607 (PMC5619807; doi:10.1371/journal.pone.0185607)
Supplement: S2 Table — (PDF) [file pone.0185607.s006.pdf]

Table a. Clinical and histopathological characteristics of the LNN subgroup of the MATCH cohort.

				SYK(T)		*SYK(S)*		*SYK(L)*		
				median (IQR)	*P value*	*median (IQR)*	*P value*	*median (IQR)*	*P value*	*Performed test*
		n	%							
Gender	Female	78	48.8%	-4.24 (-4.60 • -3.75)	0.07	-4.71 (-5.43 • -4.13)	0.09	-4.70 (-5.13 • -4.13)	0.17	*Mann-Whitney U*
	Male	82	51.3%	-3.97 (-4.61 • -3.42)		-4.48 (-5.14 • -3.82)		-4.56 (-5.14 • -3.91)		
Age		160	100%	-1.12	0.14	0.11	0.17	0.11	0.17	*Spearman's Rho*
Tumor stage	Stage I	60	37.5%	-4.31 (-4.71 • -3.67)	0.036	-4.62 (-5.21 • -3.96)	0.92	-4.73 (-5.26 • -4.28)	0.012	*Jonckheere-Terpstra*
	Stage II	100	62.5%	-3.96 (-4.55 • -3.47)		-4.61 (-5.36 • -4.07)		-4.54 (-5.04 • -3.95)		
	Stage III	-	-	-		-		-		
T status	T2	60	37.5%	-4.31 (-4.71 • -3.67)	0.036	-4.62 (-5.21 • -3.96)	0.92	-4.73 (-5.26 • -4.28)	0.012	*Mann-Whitney U*
	T3	100	62.5%	-3.96 (-4.55 • -3.47)		-4.61 (-5.36 • -4.07)		-4.54 (-5.04 • -3.95)		
Nodal status	N0	131	81.9%	-4.08 (-4.60 • -3.62)	0.60	-4.63 (-5.32 • -4.08)	0.39	-4.62 (-5.13 • -4.04)	0.98	*Jonckheere-Terpstra*
	Nx	29	18.1%	-3.81 (-4.66 • -3.43)		-4.30 (-5.28 • -3.74)		4.68 (-5.17 • -3.90)		
	N1	-	-	-		-		-		
	N2	-	-	-		-		-		
Tumor grade	Good	13	8.1%	-3.85 (-4.52 • -3.40)	0.55	-4.70 (-5.45 • -3.77)	0.42	-4.58 (-4.80 • -3.68)	0.51	*Jonckheere-Terpstra*
	Moderate	135	84.4%	-4.12 (-4.60 • -3.58)		-4.54 (-5.20 • -4.05)		-4.66 (-5.14 • -4.01)		
	Poor	9	5.6%	-4.07 (-4.71 • -3.69)		-5.37 (-5.79 • -4.09)		-4.75 (-5.16 • -3.94)		
	Other[a]	3	1.9%	-		-		-		
Location	Right	82	51.3%	-4.16 (-4.80 • -3.58)	0.16	-4.79 (-5.45 • -4.15)	0.015	-4.77 (-5.18 • -4.00)	0.14	*Mann-Whitney U*
	Left	78	48.8%	-4.04 (-4.50 • -3.52)		-4.37 (-5.10 • -3.79)		-4.58 (-4.94 • -4.03)		
MSI status [b]	MSI	37	23.1%	-4.59 (-5.11 • -4.21)	<0.001	-5.31 (-5.58 • -4.77)	<0.001	-4.99 (-5.58 • -4.54)	<0.001	*Mann-Whitney U*
	MSS	122	76.3%	-3.94 (-4.47 • -3.45)		-4.29 (-5.06 • -3.82)		-4.56 (-4.99 • -3.96)		

[a] there were no events in this subgroup
[b] n=1 missing

Table b. Clinical and histopathological characteristics of the LNP subgroup of the MATCH cohort.

				SYK(T)		*SYK(S)*		*SYK(L)*		
				median (IQR)	*P value*	*median (IQR)*	*P value*	*median (IQR)*	*P value*	*Performed test*
		n	%							
Gender	Female	34	42.5%	-4.20 (-4.60 • -3.59)	0.79	-5.07 (-5.75 • -4.14)	0.83	-4.79 (-5.37 • -4.30)	0.97	*Mann-Whitney U*
	Male	46	57.5%	-4.12 (-4.52 • -3.70)		-4.92 (-5.69 • -4.22)		-4.84 (-5.27 • -4.38)		
*Age**		80	100%	0.05	0.65	0.11	0.31	0.11	0.31	*Spearman's Rho*
Tumor stage	Stage I	-	-	-		-		-		*Jonckheere-Terpstra*
	Stage II	-	-	-		-		-		
	Stage III	80	100%	-4.15 (-4.58 • -3.68)	-	-4.98 (-5.70 • -4.19)	-	-4.81 (-5.30 • -4.33)	-	
T status	T2	11	13.8%	-4.34 (-4.63 • -3.76)	0.42	-4.69 (-5.30 • -4.11)	0.56	-5.04 (-5.70 • -4.68)	0.08	*Mann-Whitney U*
	T3	69	86.3%	-4.13 (-4.58 • -3.64)		-5.02 (-5.72 • -4.21)		-4.77 (-5.20 • -4.30)		
Nodal status	N0	-	-	-		-		-		
	Nx	-	-	-		-		-		
	N1	53	66.3%	-4.09 (-4.48 • -3.64)	0.38	-4.89 (-5.68 • -4.21)	0.67	-4.77 (-5.34 • -4.38)	0.86	*Jonckheere-Terpstra*
	N2	27	33.8%	-4.30 (-4.60 • -3.69)		-5.08 (-5.74 • -4.03)		-4.96 (-5.21 • -4.31)		
Tumor grade	Good	7	8.8%	-4.16 (-4.34 • -3.85)	0.98	-4.52 (-5.23 • -4.11)	0.68	-4.68 (-5.01 • -4.29)	0.71	*Jonckheere-Terpstra*
	Moderate	57	71.3%	-4.16 (-4.59 • -3.65)		-5.09 (-5.75 • -4.26)		-4.91 (-5.44 • -4.37)		
	Poor	11	13.8%	-4.14 (-4.58 • -3.76)		-4.89 (-6.65 • -4.33)		-4.80 (-5.36 • -4.19)		
	Other	5	6.3%	-3.67 (-4.62 • -3.35)		-3.87 (-6.41 • -3.71)		-4.46 (-4.82 • -3.88)		
Location	Right	39	48.8%	-4.29 (-4.71 • -3.72)	0.020	-5.25 (-5.99 • -4.46)	0.07	-5.09 (-5.53 • -4.48)	0.01	*Mann-Whitney U*
	Left	41	51.3%	-3.95 (-4.38 • -3.51)		-4.70 (-5.35 • -4.09)		-4.65 (-5.00 • -4.24)		
MSI status	MSI	12	15.0%	-4.59 (-4.80 • -4.28)	0.002	-5.72 (-6.57 • -5.29)	0.00	-5.19 (-5.34 • -4.59)	0.15	*Mann-Whitney U*
	MSS	68	85.0%	-4.02 (-4.46 • -3.56)		-4.71 (-5.41 • -4.02)		-4.76 (-5.19 • -4.31)		
