# Supplementary material for: High mRNA expression of splice variant SYK short correlates with hepatic disease progression in chemonaive lymph node negative colon cancer patients
Source: PLoS One. 2017 Sep 28;12(9):e0185607. doi: 10.1371/journal.pone.0185607 (PMC5619807; doi:10.1371/journal.pone.0185607)
Supplement: S3 Table — (PDF) [file pone.0185607.s007.pdf]

S3 Table. The association between epithelial and mesenchyal markers and *SYK(T)* , *SYK(S)* and *SYK(L)* for the total MATCH cohort, and the LNN and LNP subgroups of the MATCH cohort.

	EPCAM		BGN		FAP		INHBA	
	r_s	P value	r_s	P value	r_s	P value	r_s	P value
Total MATCH cohort								
SYK(T)	0.47	<0.001	0.00	0.97	-0.08	0.22	-0.01	0.86
SYK(S)	0.58	<0.001	-0.06	0.40	-0.13	0.046	-0.07	0.31
SYK(L)	0.41	<0.001	0.12	0.07	0.03	0.65	0.08	0.24
LNN cohort								
SYK(T)	0.47	<0.001	0.02	0.83	-0.06	0.46	-0.02	0.80
SYK(S)	0.55	<0.001	0.02	0.80	-0.06	0.43	-0.05	0.53
SYK(L)	0.36	<0.001	0.10	0.20	0.03	0.75	0.04	0.60
LNP cohort								
SYK(T)	0.51	<0.001	-0.01	0.91	-0.13	0.27	0.00	0.99
SYK(S)	0.59	<0.001	-0.21	0.07	-0.24	0.031	-0.17	0.14
SYK(L)	0.39	<0.001	0.16	0.16	0.06	0.58	0.12	0.29
