# Supplementary material for: High mRNA expression of splice variant SYK short correlates with hepatic disease progression in chemonaive lymph node negative colon cancer patients
Source: PLoS One. 2017 Sep 28;12(9):e0185607. doi: 10.1371/journal.pone.0185607 (PMC5619807; doi:10.1371/journal.pone.0185607)
Supplement: S4 Table — (PDF) [file pone.0185607.s008.pdf]

S4 Table. The association between infiltrate markers and *SYK(T)* , *SYK(S)* and *SYK(L)* for the total MATCH cohort, and the LNN and LNP subgroups of the MATCH cohort.

	PTPRC/CD45		VEGFA		% of infiltrating cells		% of invasive tumor cells	
	r_s	P value	r_s	P value	r_s	P value	r_s	P value
Total MATCH cohort								
SYK(T)	0.09	0.18	0.16	0.012	-0.04	0.56	-0.10	0.14
SYK(S)	0.12	0.07	0.16	0.015	-0.14	0.043	0.03	0.64
SYK(L)	0.08	0.21	0.12	0.06	0.04	0.56	-0.09	0.18
LNN cohort								
SYK(T)	0.02	0.81	0.16	0.05	-0.04	0.63	-0.10	0.19
SYK(S)	0.09	0.26	0.19	0.017	-0.24	0.007	0.04	0.59
SYK(L)	0.01	0.93	0.13	0.09	0.05	0.62	-0.14	0.07
LNP cohort								
SYK(T)	0.18	0.11	0.18	0.11	-0.03	0.80	-0.10	0.38
SYK(S)	0.10	0.38	0.09	0.42	0.02	0.87	-0.14	0.21
SYK(L)	0.08	0.48	0.10	0.39	0.07	0.57	-0.13	0.24
