# Supplementary material for: High mRNA expression of splice variant SYK short correlates with hepatic disease progression in chemonaive lymph node negative colon cancer patients
Source: PLoS One. 2017 Sep 28;12(9):e0185607. doi: 10.1371/journal.pone.0185607 (PMC5619807; doi:10.1371/journal.pone.0185607)
Supplement: S5 Table — (PDF) [file pone.0185607.s009.pdf]

Table a. Univariate cox regression analysis for the total MATCH cohort.

				DFS (events=48)		HFS (events=19)		OS (events=42)	
				HR (95%CI)	P value	HR (95%CI)	P value	HR (95%CI)	P value
		n	%						
mRNA expression	*SYK(T)*	240	100%	1.10 (0.74 • 1.64)	0.62	1.57 (0.86 • 2.88)	0.14	0.78 (0.50 • 1.22)	0.28
	SYK(S)	240	100%	1.07 (0.82 • 1.41)	0.61	1.57 (0.97 • 2.54)	0.07	0.87 (0.67 • 1.14)	0.32
	SYK(L)	240	100%	0.98 (0.66 • 1.43)	0.90	1.26 (0.70 • 2.26)	0.43	0.80 (0.52 • 1.24)	0.31
Gender	Female	112	46.7%	1		1		1	
	Male	128	53.3%	1.25 (0.70 • 2.21)	0.45	1.22 (0.49 • 3.04)	0.66	2.26 (1.15 • 4.41)	0.017
Age		240	100%	1.0002 (0.96 • 1.04)	0.99	1.001 (0.95 • 1.06)	0.96	1.06 (1.02 • 1.10)	0.004
Tumor stage	Stage I	60	25.0%	1		1		1	
	Stage II	100	41.7%	1.36 (0.59 • 3.13)	0.47	1.21 (0.36 • 4.02)	0.76	1.11 (0.47 • 2.61)	0.82
	Stage III	80	33.3%	2.25 (1.002 • 5.06)	0.049	1.42 (0.42 • 4.87)	0.57	1.90 (0.83 • 4.34)	0.13
T status	T2	71	29.6%	1		1		1	
	T3	169	70.4%	1.93 (0.93 • 3.98)	0.08	1.23 (0.45 • 3.43)	0.69	1.94 (0.89 • 4.19)	0.09
Nodal status	N0	131	54.6%	1		1		1	
	Nx	29	12.1%	1.15 (0.43 • 3.05)	0.78	3.41 (1.08 • 10.74)	0.04	1.53 (0.60 • 3.89)	0.37
	N1	53	22.1%	1.20 (0.57 • 2.55)	0.64	1.82 (0.58 • 5.72)	0.31	1.003 (0.42 • 2.42)	0.99
	N2	27	11.3%	3.58 (1.76 • 7.29)	<0.001	1.71 (0.36 • 8.26)	0.50	4.42 (2.10 • 9.27)	<0.001
Tumor grade	Good	20	8.3%	1		1		1	
	Moderate	192	80.0%	0.71 (0.28 • 1.80)	0.47	0.71 (0.16 • 3.12)	0.65	1.67 (0.40 • 6.97)	0.48
	Poor	20	8.3%	1.37 (0.43 • 4.31)	0.59	1.50 (0.25 • 8.97)	0.66	4.18 (0.89 • 19.70)	0.07
	Other[a]	8	3.3%	-	-	-	-	-	-
Location	Right	121	50.4%	1		1		1	
	Left	119	49.6%	1.19 (0.68 • 2.11)	0.54	0.91 (0.37 • 2.24)	0.84	0.58 (0.31 • 1.08)	0.09
MSI status [b]	MSI	49	20.4%	1		1		1	
	MSS	190	79.2%	2.32 (0.92 • 5.85)	0.08	29.05 (0.26 • 3292.13)	0.16	0.67 (0.34 • 1.32)	0.25

[a] there were no events in this subgroup
[b] n=1 missing

Table b. Univariate cox regression analysis for the LNN subgroup of the MATCH cohort.

		n	%	DFS (events=26) HR (95%CI)	P value	HFS (events=12) HR (95%CI)	P value	OS (events=23) HR (95%CI)	P value
mRNA expression	*SYK(T)*	160	100%	1.33 (0.81 • 2.19)	0.26	2.05 (1.01 • 4.17)	0.047	0.80 (0.45 • 1.42)	0.44
	SYK(S)	160	100%	1.35 (0.89 • 2.04)	0.16	2.14 (1.14 • 4.01)	0.018	0.91 (0.62 • 1.32)	0.60
	SYK(L)	160	100%	1.02 (0.62 • 1.68)	0.95	1.44 (0.72 • 2.87)	0.31	0.78 (0.44 • 1.39)	0.39
Gender	Female	78	48.8%	1		1		1	
	Male	82	51.3%	1.62 (0.73 • 3.56)	0.24	1.97 (0.59 • 6.53)	0.27	2.33 (0.96 • 5.68)	0.06
Age		160	100%	1.002 (0.96 • 1.05)	0.94	1.01 (0.94 • 1.08)	0.79	1.08 (1.02 • 1.14)	0.011
Tumor stage	Stage I	60	37.5%	1		1		1	
	Stage II	100	62.5%	1.36 (0.59 • 3.12)	0.47	1.21 (0.36 • 4.02)	0.76	1.10 (0.47 • 2.61)	0.82
	Stage III	-	-	-	-	-	-	-	-
T status	T2	60	37.5%	1		1		1	
	T3	100	62.5%	1.36 (0.59 • 3.12)	0.47	1.21 (0.36 • 4.02)	0.76	1.10 (0.47 • 2.61)	0.82
Nodal status	N0	131	81.9%	1		1		1	
	Nx	29	18.1%	1.16 (0.44 • 3.07)	0.77	3.42 (1.09 • 10.78)	0.036	1.52 (0.60 • 3.87)	0.38
	N1	-	-	-	-	-	-	-	-
	N2	-	-	-	-	-	-	-	-
Tumor grade	Good	13	8.1%	1		1		1	
	Moderate	135	84.4%	1.02 (0.24 • 4.36)	0.98	0.88 (0.11 • 6.91)	0.27	1.92 (0.26 • 14.38)	0.52
	Poor	9	5.6%	2.14 (0.36 • 12.82)	0.40	2.85 (0.26 • 31.39)	0.39	4.47 (0.46 • 43.15)	0.20
	Other[a]	3	1.9%	-	-	-	-	-	-
Location	Right	82	51.3%	1		1		1	
	Left	78	48.8%	2.06 (0.92 • 4.62)	0.08	1.09 (0.35 • 3.38)	0.88	0.60 (0.26 • 1.42)	0.25
MSI status [b]	MSI	37	23.1%	1		1		1	
	MSS	122	76.3%	3.90 (0.92 • 16.49)	0.07	31.28 (0.12 • 8430.24	0.23	0.51 (0.22 • 1.17)	0.11

[a] there were no events in this subgroup

[b] n=1 missing

Table c. Univariate cox regression analysis for the LNP subgroup of the MATCH cohort.

				DFS (events=22) HR (95%CI)	P value	HFS (events=7) HR (95%CI)	P value	OS (events=19) HR (95%CI)	P value
		n	%						
mRNA expression	*SYK(T)*	80	100%	0.78 (0.39 • 1.56)	0.48	0.78 (0.23 • 2.67)	0.69	0.71 (0.33 • 1.52)	0.37
	SYK(S)	80	100%	0.95 (0.65 • 1.37)	0.77	1.08 (0.54 • 2.16)	0.83	0.90 (0.60 • 1.34)	0.59
	SYK(L)	80	100%	1.08 (0.56 • 2.07)	0.82	1.02 (0.32 • 3.27)	0.98	0.95 (0.46 • 1.94)	0.88
Gender	Female	34	42.5%	1		1		1	
	Male	46	57.5%	0.85 (0.37 • 1.96)	0.70	0.54 (0.12 • 2.41)	0.42	1.96 (0.70 • 5.48)	0.20
Age		80	100%	1.03 (0.96 • 1.10)	0.37	0.99 (0.88 • 1.12)	0.89	1.10 (1.02 • 1.18)	0.01
Tumor stage	Stage I	-	-	-		-		-	
	Stage II	-	-	-		-		-	
	Stage III	80	100%	-		-		-	
T status	T2	11	13.8%	1		1		1	
	T3	69	86.3%	3.84 (0.52 • 28.57)	0.19	1.08 (0.13 • 8.97)	0.94	26.85 (0.14 • 5108.71)	0.22
Nodal status	N0	-	-	-	-	-		-	
	Nx	-	-	-	-	-		-	
	N1	53	66.3%	1		1		1	
	N2	27	33.8%	2.85 (1.23 • 6.60)	0.015	0.93 (0.18 • 4.81)	0.93	4.14 (1.63 • 10.57)	0.003
Tumor grade	Good	7	8.8%	1		1		1	
	Moderate	57	71.3%	0.56 (0.16 • 1.92)	0.35	0.58 (0.07 • 4.93)	0.62	1.43 (0.19 • 10.98)	0.73
	Poor	11	13.8%	0.82 (0.18 • 3.66)	0.79	0.63 (0.04 • 10.01)	0.74	3.12 (0.36 • 26.75)	0.30
	Other[a]	5	6.3%	-	-	-	-	-	-
Location	Right	39	48.8%	1		1		1	
	Left	41	51.3%	0.60 (0.26 • 1.40)	0.24	0.65 (0.15 • 2.91)	0.57	0.52 (0.20 • 1.31)	0.17
MSI status	MSI	12	15.0%	1		1		1	
	MSS	68	85.0%	0.995 (0.29 • 3.36)	0.99	25.07 (0.002 • 332856.52)	0.51	0.86 (0.25 • 2.98)	0.81

[a] there were no events in this subgroup
